# Secondary prevention of allergic symptoms in a dairy farmer by use of a milking robot

**DOI:** 10.1186/1476-7961-3-8

**Published:** 2005-06-22

**Authors:** Gintautas Korinth, Horst Christoph Broding, Wolfgang Uter, Hans Drexler

**Affiliations:** 1 Institute and Outpatient Clinic of Occupational, Social and Environmental Medicine, University Erlangen-Nuremberg, Schillerstrasse 25/29, D-91054 Erlangen, Germany; 2 Department of Medical Informatics, Biometry and Epidemiology, University Erlangen-Nuremberg, Waldstrasse 6, D-91054 Erlangen, Germany

## Abstract

**Background:**

Animal-derived allergens include lipocalins which play an increasing role in occupational respiratory sensitizations. The prevention of sensitization in stock farming is often difficult due to intense exposure, with traditional milking still requiring close animal contact. Complete avoidance of allergen exposure is only possible if stock farming is abandoned. This is, however, often not feasible in small dairy plants because of the resulting loss of income.

**Case presentation:**

In a 37-year-old female farmer daily asthmatic complaints appeared, associated with cow dust-derived allergen exposure by milking with a conventional device. Respiratory symptoms increased during a period of 12 years. Allergic bronchial asthma was diagnosed, caused by sensitization against cow dust-derived allergens, as demonstrated by positive skin prick test and by detection of IgE antibodies. In a separate specific inhalation challenge test using a 10% extract of cow dust-derived allergens a 330% increase of airway resistance was detected. To enable further dairy farming, a milking robot was installed in 1999, i.e., an automatic milking system. The novel milking technique reduced the daily exposure from over 2 hours to approximately 10 min. The clinical course after the installation of the milking robot was favourable, with less frequent allergic and asthmatic symptoms. Furthermore, asthma medication could be reduced. Improvement was noted also in terms of lung-function and decreased total serum IgE.

**Conclusion:**

The case presented and the evidence from the literature indicates that the strategy of exposure minimization to allergens at workplaces can be an effective alternative to total elimination. In farmers with cow dust allergy a milking robot is an appropriate technical measure to minimize allergen-exposure.

## Background

Allergic asthma ranks in Germany among frequent occurred occupational diseases. Cow hair allergy in Finland is one of the most frequent reasons for the development of occupational asthma [1], but epidemiologically in Germany still insufficiently explored. The prevalence of respiratory complaints in Finnish farmers with cow husbandry is 27% for rhinitis respectively 12% for asthma in consequence of occupational exposure [2,3]. Until now there is only limited insight into the risk factors with regard to this disease. The prevention of sensitization in stock farming to cow hair and epithelial allergens including lipocalins is often difficult due to intense exposure, with traditional milking still requiring close animal contact.

The German Professionals insurance laws require a complete elimination of allergens for the recognition of an allergic airway disease as a compensable occupational disease. Complete avoidance of occupational exposure of cow hair allergens is only possible if stock farming is abandoned. This is, however, often not feasible in small dairy plants because of the resulting loss of income.

In the present case report we discuss the efficiency of the strategy of exposure minimization to allergens by the use of a milking robot, i.e., an automatic milking system (AMS) as an alternative measure to total elimination of allergens for the prevention of occupational asthma caused by cow hair allergy.

## Case presentation and discussion

A 37-year-old female farmer reported to suffer from daily asthmatic complaints by milking of cows with a conventional device. Exposure associated respiratory symptoms increased continuously during a period of 12 years. Allergic bronchial asthma, caused by sensitization against cow dust-derived allergens, was diagnosed by a positive skin prick test (Bencard^®^) and by the detection of IgE antibodies against *Bos d 2 *at 74.9 kIU/l (Pharmacia, UniCAP^®^). Moreover, nonspecific bronchial hyperresponsiveness was verified by a positive histamine inhalation challenge test. The diagnosis of occupational asthma was confirmed by a separate specific inhalation challenge test using a 10% extract of cow hair allergens which led to a 330% increase of airway resistance (Raw).

The German Professionals insurance laws require complete elimination of allergen exposures for the compensation of occupational allergic asthma as occupational disease [4]. Legal consequence would usually be to abandon dairy farming. However, in our case presented, dairy farming substantially contributed to family-income and the compensation offered would be insufficient for subsistence. Hyposensitization is the most effective measure in the prevention of allergic symptoms in patients with asthma. In that farmer the hyposensitization was not feasible due to permanent exposure to allergens. This farmer did not wear a helmet during the milking process because of claustrophobic panic attacks (fear of suffocation). Moreover an immunotherapy with recombinant lipocalins is currently not available.

Respirator helmets have been predominantly used as effective measures for prevention of the inhalation of fumes and dusts [5]. Investigations about the effectiveness of respirator helmets against high-molecular allergens to prevent allergic asthma are rare. Taivainen et al. (1998) demonstrated that due to the use of a dust respirator helmet with a P2-class filter, dairy farmers with occupational asthma induced by cow dander showed less rhinitic symptoms and an improved peak flow rate [6]. However, workers using respirator helmets were not prevented against a progressive behavior of occupational asthma [7,8]. Thus it is to doubt whether respirator helmets are efficient in farmers with occupational asthma to provide a complete protection against allergens [9].

**Figure 1:**
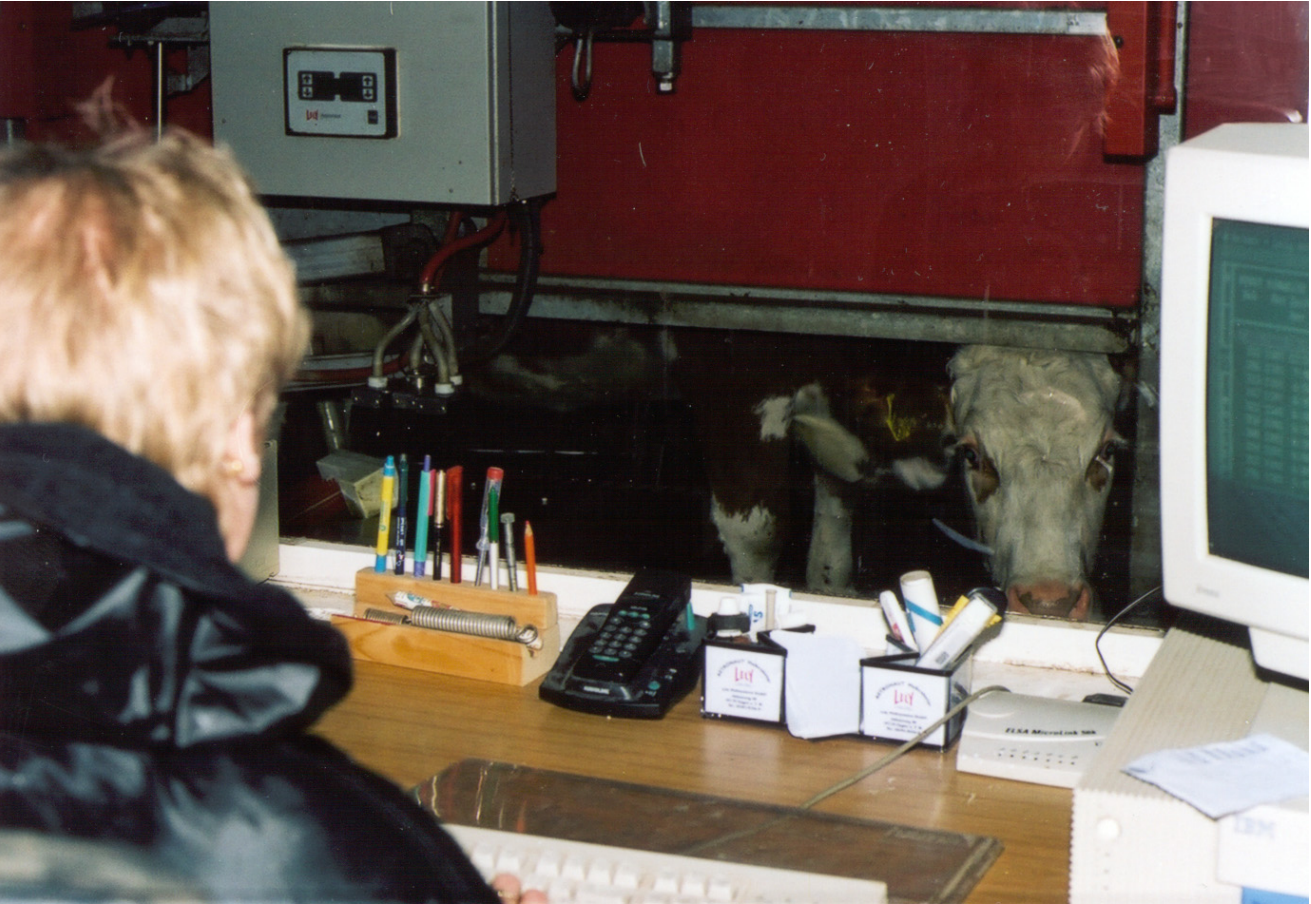
Milking process monitoring by an air-shielded control-centre.

To enable further dairy farming, the farmer installed a milking robot in 1999, i.e., an automatic milking system (AMS). At present circa 1300 AMS are installed, predominantly in the Netherlands, followed by Germany. AMS were developed in the course of increasing automation of the agricultural production. AMS consist of a milking box with the milking equipment and an electronic controlling system, an automatically operating dosing unit of a specific food-concentrate as well as a software controlled management system. The cows are decoyed from a specific food-concentrate to enter the milking box. A transponder, fixed on the cow neck, initiates milking operations automatically controlled by software and laser. The milking robot operates in the cowshed, which is air-shielded from the control-centre. After the installation of AMS the farmer was involved only in terms of monitoring the milking process. Figure 1 shows the job and the current exposure situation of the farmer. It is apparent that the novel milking technique reduced exposure significantly by some 90% (from over 2 hours to approx. 10 min.) without a decline of milk production. Beyond allergen exposure reduction, the physical workload was lessened and the time available for activities to improve coping was increased – these factors are increasingly recognized as relevant for the prognosis of asthma [10]. Table 1 shows the influence of the AMS technique on the current occupational exposure of the farmer. According to German Professionals insurance laws all conceivable measures must be taken into account even AMS is an expensive technical device to prevent occupational asthma.

**Table 1:** Parameters influenced by automatic milking system (AMS)

**Parameter**	**Power of parameter**	**Influence of AMS**
Daily exposure period	↓↓↓	Drop of more than 2 hours to approx. 10 min. (> 90%)
Exposure intensity	↓↓↓	Increasing space to cow
Physical workload	↓↓	Monitoring tasks
Timing flexibility	↑↑	• No commitment to fixed milking times • Free working plan
Quality of life	↑	More time available for other activities

Explanation: ↓ = reduction of power, ↑ = improvement of power

After installation of the milking robot the farmer reported significantly less frequent allergic and asthmatic symptoms. Asthma medication was reduced and required only once daily in the evening. Improvement was noted also in terms of lung-function and decreased total serum IgE: serum IgE decreased from 1332 IU/ml before milking robot installation to 572 and 474 IU/ml two and three years after installation, respectively. FEV_1 _improved by 20% and vital capacity by 11%, comparing values one year before with two years after installation. A CAP-RAST score "5" versus *Bos d 2 *was determined after the intervention. Preceding EAST-Test results vs. *Bos d 2 *were not suitable for comparison.

Current discussions address threshold limit values for allergens that may prevent allergic symptoms [11]. These have been proposed also for the cow dust-derived allergen *Bos d 2 *[12]. Table 2 shows examples for successful measures of exposure minimization at workplaces to reduce sensitizations. Recent studies have already demonstrated that allergen exposure reduction at workplaces is successful in reducing sensitizations, e.g., to latex proteins [13,14], laboratory animals [15] or acid anhydrides [16].

**Table 2:** Examples for successful measures of exposure minimization at workplaces in reducing of sensitizations

**Exposure areas **	**Allergen**	**Measure of exposure minimization**	**Study**
Hospitals	Latex proteins	Replacement of powdered gloves through powder-free gloves	Edelstam et al. 2002, Tarlo et al. 1994
Animal research laboratories	Allergens to laboratory animals	Technical improvement of housing cabinets for animals	Thulin et al. 2002
Electrical industry	Acid anhydrides	Installation of a closed system in the production of epoxy resin	Drexler et al. 1999

## Conclusion

The case presented and the evidence obtained from the literature indicates that the strategy of exposure minimization to allergens at workplaces can be an effective alternative to total elimination, which may not be feasible for economical reasons. In farmers with cow dust allergy a milking robot is an appropriate technical measure to minimize allergen-exposure particularly with regard to the reduced animal contact. Independently from this, such milking robots also increasingly used to enhance productivity (usually in herds comprising more than 50 animals). The efficacy of milking robots in terms of primary and secondary prevention should be evaluated also epidemiologically in a suitable cohort study, preferably in countries with intensive dairy farming.

## List of abbreviation

AMS; Automatic milking system

## Competing interests

The author(s) declare that they have no competing interests.

## Authors' contributions

GK was the principal investigator. GK examined the patient, performed the investigations and drafted the manuscript. HCB revised the manuscript. WU revised the manuscript and evaluated the epidemiological aspects. HD conceived the case report. All authors read and approved the final manuscript.
